# Genetic Diversity and Antiretroviral Resistance in HIV-1-Infected Patients Newly Diagnosed in Cabo Verde

**DOI:** 10.3390/v16121953

**Published:** 2024-12-20

**Authors:** Silvânia Da Veiga Leal, Victor Pimentel, Paloma Gonçalves, Isabel Inês Monteiro de Pina Araújo, Ricardo Parreira, Nuno Taveira, Marta Pingarilho, Ana B. Abecasis

**Affiliations:** 1 Instituto Nacional de Saúde Pública de Cabo Verde, Largo do Desastre da Assistência, Chã de Areia, Praia CP 7943-010, Cape Verde; 2 Global Health and Tropical Medicine, GHTM, Associate Laboratory in Translation and Innovation Towards Global Health, LA-REAL, Instituto de Higiene e Medicina Tropical, IHMT, Universidade NOVA de Lisboa, Rua da Junqueira 100, 1349-008 Lisboa, Portugal; 3 Research Institute for Medicines (iMed.ULisboa), Faculdade de Farmácia, Universidade de Lisboa, Avenida Professor Gama Pinto, 1649-003 Lisboa, Portugal; 4 Faculdade de Ciências e Tecnologia, Universidade de Cabo Verde, Campus do Palmarejo Grande, Praia CP 7943-010, Cape Verde; 5 One Health Research Center—Cabo Verde (NEST-CV), Universidade de Cabo Verde, Campus do Palmarejo Grande, Praia CP 7943-010, Cape Verde; 6 Centro de Investigação Interdisciplinar Egas Moniz (CiiEM), Egas Moniz School of Health & Science, Quinta da Granja, Monte de Caparica, 2829-511 Almada, Portugal

**Keywords:** transmitted drug resistance, HIV-1 subtypes, genetic diversity, genomic surveillance, Cabo Verde

## Abstract

The high genetic variability of HIV-1 and the emergence of transmitted drug resistance (TDR) can impact treatment efficacy. In this study, we investigated the prevalent HIV-1 genotypes and drug-resistance-associated mutations in drug-naïve HIV-1 individuals in Cabo Verde. The study, conducted between 2018 and 2019, included drug-naïve HIV-1 individuals from the São Vicente, Boa Vista, Fogo, and Santiago islands. The HIV-1 *pol* gene was sequenced using Sanger sequencing. TDR was identified using the Stanford Calibrated Population Resistance tool, and resistance levels to different drugs were interpreted with the Stanford HIV database. The genetic diversity of HIV-1 was determined through phylogenetic analysis, and epidemiological and behavioural data were collected via questionnaires. Of the 73 participants, the majority were male (52.1%). The CRF02_AG recombinant form predominated (41.1%), followed by subtype G (37.0%). The overall prevalence of TDR was 9.6%. Nucleoside Reverse Transcriptase Inhibitor (NRTI) mutations occurred in 2.7% of individuals, while Non-Nucleoside Reverse Transcriptase Inhibitor (NNRTI) mutations occurred in 9.6%. The most prevalent mutations were K103N (5.5%) and M184V (2.7%). No protease- or integrase-associated mutations were found. The high levels of resistance to NNRTIs found demonstrate the need for surveillance of resistance mutations to ensure the efficacy and durability of the current therapeutic regimen, which includes Dolutegravir.

## 1. Introduction

Human immunodeficiency virus (HIV) infection remains a major global public health issue. By the end of 2022, approximately 39 million people were living with HIV, with 1.3 million new infections and 29.8 million people receiving antiretroviral therapy (ART) [1,2]. ART has significantly reduced morbidity and mortality in people living with HIV [3]. However, the high genetic variability of HIV-1 can lead to the selection of antiretroviral drug resistance mutations [4]. Drug-resistant HIV variants can be transmitted to treatment-naïve individuals and further spread throughout the population over time [5,6]. Transmitted drug resistance (TDR) results from infection with an HIV-1 strain containing one or more resistance-associated mutations [7,8]. Several studies have described the prevalence of TDR in treatment-naïve patients, which varies by geographic region. Studies in the United States and Europe have shown that the prevalence of TDR ranges from 5 to 15% [9,10,11,12]. However, in countries with limited resources, where data are scarce, some reports describe an exponential increase in TDR prevalence, and many exceed 10% of TDR prevalence [13].

Cabo Verde is an archipelago of ten islands, located in the Atlantic Ocean, 445 km from the coast of West Africa, with a population of approximately 491,233 [14]. The prevalence of HIV-1 infection was about 0.6% in 2018, with higher rates among women (0.7%) than among men (0.3%) [15]. HIV-1 is the predominant type of HIV in the country, with a prevalence of 0.46%, being significantly more common than HIV-2, which has a much lower prevalence [16].

ART in Cabo Verde began in 2004, following the recommendations of the World Health Organization (WHO). Since then, antiviral medications have been provided free of charge, and treatment is initiated as early as possible following an HIV infection diagnosis [17,18]. In recent years, HIV treatment guidelines have been significantly updated, reflecting ongoing advances in the field [18,19,20,21]. In 2018, WHO introduced dolutegravir (DTG) as the preferred choice in first- and second-line therapeutic regimens for people living with HIV-1. This change was made due to the increasing resistance to Non-Nucleoside Reverse Transcriptase Inhibitors (NNRTIs), which were replaced by DTG [21,22]. The most recent national guidelines for ART in adults and adolescents living with HIV-1 in Cabo Verde date from 2019 [17] and incorporate WHO recommendations, including the initiation of antiretroviral therapy for all people living with HIV (PLHIV), regardless of CD4 cell count. Additionally, the guidelines address ideal treatment regimens and the management of HIV coinfection with opportunistic infections. Accordingly, DTG was introduced as the first-line drug in a regimen combining two Nucleoside Reverse Transcriptase Inhibitors (NRTIs) and one integrase strand transfer inhibitor (INSTI) [21].

The data on the molecular epidemiology of drug resistance mutations (DRMs) and HIV-1 genotypes circulating in Cabo Verde are limited. However, some studies have already identified HIV-1 subtypes such as G, F, B, C, and A, as well as recombinant forms CRF02_AG, CRF01_AG, CRF02_AE, and CRF05_DF and complex recombinant forms. These studies also report that DRMs were also identified in the protease (PR) and Reverse Transcriptase (RT) of treated and naïve patients [23,24,25]. Previous studies involving untreated patients are outdated, with the most recent research focusing exclusively on treated individuals. This highlights the need for updated research on HIV-1 genotypes and resistance profiles. Our study aims to address this gap by investigating the most prevalent HIV-1 genotypes and drug resistance mutations in newly diagnosed and drug-naïve individuals in Cabo Verde.

## 2. Materials and Methods

### 2.1. Study Population and Sample Collection

Drug-naïve individuals diagnosed with HIV-1 between 2018 and 2019 on 4 different islands of Cabo Verde (São Vicente, Boa Vista, Fogo, and Santiago) were included in this study. A semi-structured questionnaire was used to collect data on demographic, socioeconomic, behavioural, and clinical characteristics of the patients through face-to-face interviews and from the participants’ medical records. Written informed consent was obtained from all participants after informing respondents of the aim of the study, potential risks and benefits of participation, and the rights of the participants. Blood samples were collected in EDTA tubes, and aliquots of plasma were stored at −80 °C. Plasma samples were anonymized and transferred to Portugal for genotyping by population sequencing.

### 2.2. HIV Subtyping and Drug-Resistance Mutation Analyses

Viral RNA was extracted from HIV-1 plasma samples using the commercial kit QIAamp^®^ Viral RNA Mini KIT (Qiagen, Hilden, Germany), according to the manufacturer’s instructions. One-step reverse-transcription PCR (one-step RT-PCR) was performed for the PR, RT, and IN regions of the polymerase gene using the SuperScript III One-Step RT-PCR System with the PlatinumTM Taq DNA Polymerase kit from InvitrogenTM (Thermo Fisher Scientific, Carlsbad, CA, USA), following the manufacturer’s protocol. Subsequently, nested PCR was conducted using the NZYTaq II DNA polymerase kit (NZYtech genes & enzymes, Lisbon, Portugal), following the manufacturer’s recommendations. Genotyping of the HIV-1 *pol* gene was performed using primers previously described [25]. The PCR products were purified using the NucleoSpinR Gel and PCR Clean-Up (Macherey-Nagel, Düren, Alemanha) following the manufacturer’s protocol, and the nucleotide sequencing of the purified DNA fragments was performed by STAB VIDA (Oeiras, Portugal) by the You Tube It system, using the method described by Sanger et al. [26]. Sequence electropherograms were visualized, assembled, and aligned using the bioinformatics software Geneious Prime (version 2022.0.1, Biomatters, Auckland, New Zealand), and the resulting consensus sequences were aligned with an HIV-1 reference sequence (B-HXB2-PRT_ 2253–3700). Subtyping was performed using REGA v.3.0, Stanford HIV, and Comet software. To infer the phylogenetic relationships between the newly generated sequences, a maximum likelihood phylogenetic tree was constructed for the pol gene. The tree was constructed using all reference subtypes and subsubtypes, including the CRFs (01_AE and 02_AG). All sequences were manually aligned and adjusted using Virulign and AliView. Subsequently, a phylogenetic tree based on the general nucleotide substitution (GTR) model was constructed in FastTree (version 2.1.11). TDR mutations were identified using the Stanford Calibrated Population algorithm (https://hivdb.stanford.edu/cpr/, accessed on 28 July 2023). For measuring the clinical impact of DRMs, the Stanford Genotypic Resistance Interpretation Algorithm was used [7,11].

### 2.3. Statistical Analysis

Statistical analysis was performed with IBM SPSS 27.0 (Statistical Package for Social Sciences, Chicago, IL, USA). Data were analyzed using contingency tables with the chi-square test and Fisher’s exact test. For statistical analysis, we considered a 5% significance level. A binary logistic regression model was used to examine the association between baseline demographic, clinical, socioeconomic, and behavioural factors and the occurrence of TDR mutations. The results are presented using Odds Ratios (ORs). Proportions and confidence intervals for proportions were calculated using the 95% Wilson Confidence Interval for binomially distributed data. 

### 2.4. Ethics Approval and Consent to Participate

This study was reviewed and approved by the Ethical Committee of the Ministry of Health of Cabo Verde (Reference no. 13/2018) and by the National Data Protection Commission (reference no. 192/2018). The privacy and confidentiality issues had been secured throughout the processes.

## 3. Results

### 3.1. Study Population Demographics and Clinic Characteristics

This study included 73 drug-naïve PLHIV. When analyzing the sex distribution, we observed that 47.9% (n = 35) were female. The subtype distribution of the infecting strains in female patients was as follows: 15.1% (n = 11) belonged to the CRF02_AG recombinant form, 20.5% (n = 15) to subtype G, and 12.3% (n = 9) to other subtypes. Out of the 52.1% (n = 38) male participants, 26.0% (n = 19) were infected with the HIV-1 CRF02_AG recombinant form, 16.4% (n = 12) with subtype G, and 9.6% (n = 7) with other subtypes. The average age of the participants was 44.8 years (±14.8).

The age groups with the highest number of individuals (31–40 years and 41–50 years) were more frequently infected with the HIV-1 subtype G (13.7%; n = 10) and CRF02_AG recombinant form (12.3%; n = 9), respectively. The majority of participants resided on the island of Santiago (75.3%; n = 55), where more cases of infections with subtype G and the CRF02_AG recombinant form were found (32.9%, n = 24; 31.5%, n = 23, respectively), followed by other subtypes (11.0%, n = 8).

When analyzing participants stratified by island of residence, the island of residence was found to be significantly associated with viral subtype (*p* = 0.014). However, other demographic and clinical characteristics were not significantly associated with the viral subtypes/genetic recombinants. Within participants with TDR, the most common subtypes were the CRF02_AG recombinant form (5.5%; n = 4) and subtype G (4.4%; n = 3). Table 1 presents the demographic and clinical characteristics of the participants, stratified by the different viral subtypes.

### 3.2. Genetic Diversity of HIV-1 Subtypes

**Figure 1:**
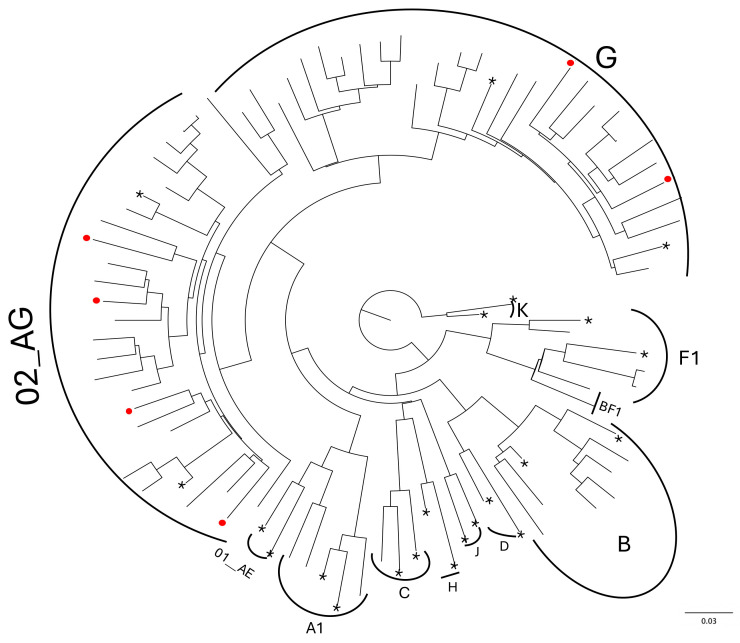
Maximum likelihood (ML) phylogenetic tree analysis of 68 HIV-1 pol region nucleotide sequences collected from 2018 to 2020 in Cabo Verde. Phylogenetic analyses were performed using a comprehensive reference data set that includes all known HIV-1 group M subtypes (A, B, C, D, F, G, H, J, K) and circulating recombinant forms (CRFs: 01AE and 02AG) (N = 22) obtained from the Los Alamos HIV Sequence Database (http://www.hiv.lanl.gov, accessed on 26 September 2024). * Taxons with asterisks represent reference sequences for each subtype. Red hatched circles represent sequences harbouring some SDRM.

In total, six different HIV-1 subtypes were identified, including five pure subtypes and one circulating recombinant form (CRF02_AG). Phylogenetic analysis revealed that most cases (41%, n = 30) were caused by CRF02_AG strains, followed by subtype G (37%, n = 27), subtype B (10%, n = 7), subtype F (7%, n = 5), subtype A (4%, n = 3), and subtype C (1%, n = 1). No pattern suggestive of the transmission of TDR was observed in the phylogenetic analysis of the distribution of sequences carrying surveillance drug resistance mutations (SDRMs). The sequences containing SDRMs are marked with red dots on the graph, with most of these mutations observed in the CRF_02AG strains and subtype G, which are the predominant strains in the country (Figure 1).

**Figure 2:**
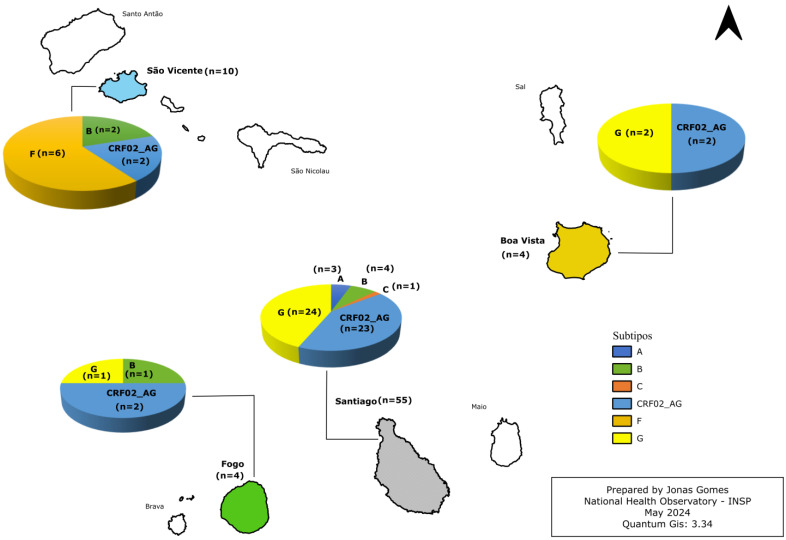
Distribution of genetic diversity of HIV-1 subtypes in the islands of Cabo Verde.

The geographic distribution of HIV-1 subtypes in Cabo Verde varies between the islands. Santiago has the highest number of cases (n = 55), with a predominance of subtype G (43.6%, n = 24) and CRF02_AG (41.8%, n = 23). In São Vicente (n = 10), subtype F is the most prevalent (60%, n = 6), while Boavista (n = 4) only presents cases of CRF02_AG (50%, n = 2) and subtype G (50%, n = 2). On the island of Fogo (n = 4), cases are distributed among subtype B (25%, n = 1), CRF02_AG (50%, n = 2), and subtype G (25%, n = 1) (Figure 2).

### 3.3. Prevalence of HIV-1-Transmitted Drug Resistance

**Figure 3:**
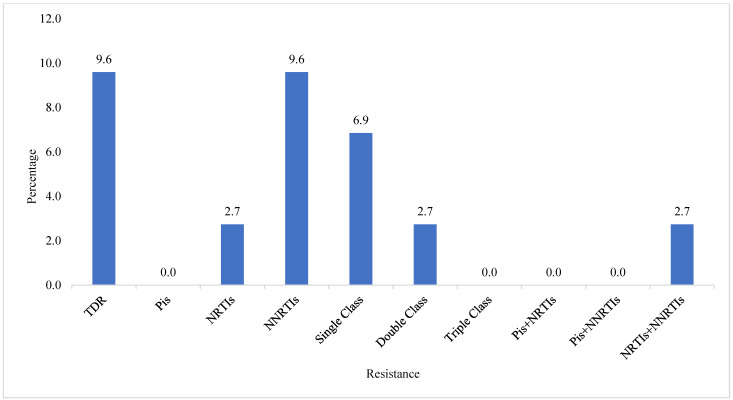
Prevalence of transmitted HIV-1 drug resistance in Cabo Verde. NRTIs: Nucleoside Reverse Transcriptase Inhibitors. NNRTIs Non-Nucleoside Reverse Transcriptase Inhibitors. TDR: transmitted HIV-1 drug resistance. PIs: protease inhibitors. NSTIs: integrase strand transfer inhibitors.

Of the 73 pol sequences analyzed, the overall prevalence of TDR was 9.6% (n = 7). Among the patients, mutations in RT conferring resistance to NRTIs were identified in 2.7% (n = 2), while resistance to NNRTIs was found in 9.6% (n = 7). No resistance mutations were found in PR and IN genes that confer resistance to protease inhibitors (PIs) or INSTIs, respectively. Single-class resistance mutations were found in 6.8% (n = 5) and dual-class resistance mutations to NRTIs + NNRTIs were found in 2.7% (n = 2) of the patients. No triple-class resistance mutations were found. No resistance was found for other combinations of drug classes (PIs + NRTIs, PIs + NNRTIs) (Figure 3).

**Figure 4:**
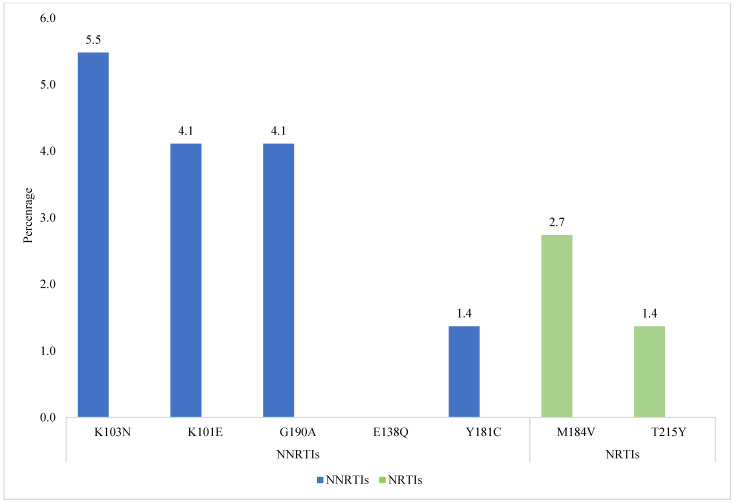
Prevalence of Non-Nucleoside Reverse Transcriptase Inhibitor (NNRTI) resistance mutations (blue) and Nucleoside Reverse Transcriptase Inhibitor (NRTI) resistance mutations (green).

Mutations were found conferring resistance to two classes of antiretroviral drugs: NNRTIs and NRTIs. For the NNRTI class, the most frequently found mutations were K103N (5.5%; 4/73), K101E (4.1%; 3/73), G190A (4.1%; 3/73), E138Q (2.7%; 2/73), and Y181C (1.4%; 1/73). For the NRTI class, the most frequent resistance mutations were M184V (2.7%; 2/73) and T215Y (1.4%; 1/73) (Figure 4).

### 3.4. Resistance Levels to Various ARV Drugs

The levels of resistance associated with each specific drug were based on the interpretation of the Stanford HIV database. The levels of resistance were classified into five categories: Susceptible (S), Potential Low-level Resistance (PLR), Low-Level Resistance (LLR), Intermediate Resistance (IR), and High-Level Resistance (HLR). For the NRTI class, the following mutations were found: M184V and M184V + T215Y. The M184V mutation confers HLR to Emtricitabine (FTC) and Lamivudine (3TC), and LLR to Abacavir (ABC). The combination of M184V + T215Y confers HLR to FTC and 3TC.

For the NNRTI class, the following mutations were found: K103N, K101E, E138Q, G190A, and Y181C. The K103N mutation confers HLR to doravirine (DOR), efavirenz (EFV), etravirine (ETR), nevirapine (NVP), and rilpivirine (RPV). The Y181C mutation causes IR to EFV, ETR, and RPV, and HLR to NVP. The combinations of mutations, namely K101E, K103N, and G190GA, result in HLR to NVP, RPV, and EFV, as well as IR to ETR and low-level resistance to DOR. Another identified combination, K103N and E138A, confers HLR to NVP and LLR to ETR and RPV. In the case of the combination K101E, E138Q, and G190A, there is HLR to NVP and RPV, IR to ETR, and LLR to DOR (Table 2).

**Figure 5:**
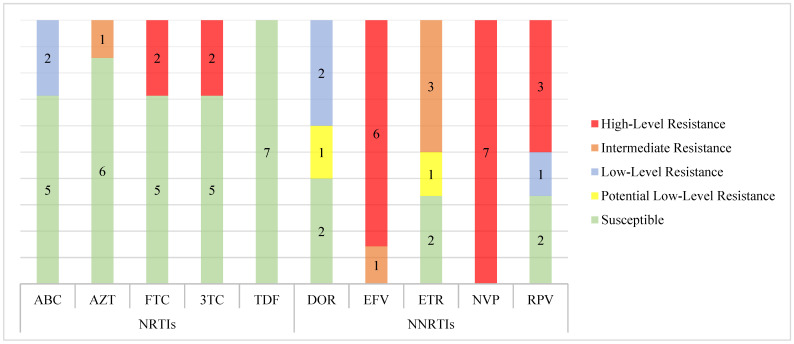
Frequency and levels of HIV-1 drug resistance to various antiretroviral drugs. NRTIs: Nucleoside Reverse Transcriptase Inhibitors (3TC: lamivudine; FTC: emtricitabine; ABC: abacavir; AZT: zidovudine; TDF: tenofovir). NNRTIs: Non-Nucleoside Reverse Transcriptase Inhibitors (NVP: nevirapine; EFV: efavirenz; ETR: etravirine; RPV: rilpivirine; DOR: doravirine). Red represents high-level resistance, orange represents intermediate-level resistance, blue represents low-level resistance, yellow represents potential low-level resistance, and green represents drug susceptibility.

Of the two sequences with mutations associated with resistance to NRTIs, both exhibited high-level resistance to 3TC and FTC, and one showed IR to zidovudine (AZT). In the case of the seven sequences with mutations associated with NNRTIs, all of them showed HLR to NVP, while six of them (85.7%) exhibited HLR to EFV. Regarding RPV, three sequences (42.9%) showed HLR. Notably, for DOR, only LLR or PLLR was identified (Figure 5).

### 3.5. Factors Associated with the Presence of Mutations Associated with Antiretroviral Resistance

None of the analyzed sociodemographic and clinical variables was significantly associated with the occurrence of TDR mutations (*p* > 0.05) (Table 3). However, TDR mutations were more common among men than women (5.5% versus 4.1%, *p* = 0.777), and participants under the age of 30 had a higher proportion of TDR compared to other age groups (5.5% vs. 2.7%, *p* = 0.273). Employed individuals had higher proportions of TDR compared to unemployed individuals and students (6.8% versus 1.4%, OR = 2.36, 95% CI 0.23–24.33, *p* = 0.471; 6.8% versus 1.4%, OR = 5.9, 95% CI 0.45–76.95, *p* = 0.176). It was observed that the majority of participants (34 out of 60, or 56.7%) had CD^4+^ T cell counts below 350 cells/mm^3^ at the time of diagnosis, indicating a late diagnosis of HIV-1 infection. Furthermore, individuals with CD^4+^ T cell counts below 350 cells/mm^3^ presented with TDR more frequently compared to those with CD^4+^ T cell counts between 350 and 499 cells/mm^3^ (5.5% vs. 1.4% OR = 0.625, 95% CI 0.063–6.180, *p* = 0.688). The prevalence of TDR was higher among participants with HIV-1 recombinant subtype CRF02_AG compared to those with subtype G (5.5% versus 4.1%, OR = 1.231, IC 95% 0.25–6.07, *p* = 0.799). The sample size in this study was small, which may have limited the detection of statistically significant differences.

## 4. Discussion

In this study, we used a genomic surveillance approach combined with sociodemographic and clinical questionnaires to characterize a population of newly diagnosed HIV-1-infected drug-naïve individuals from Cabo Verde, including individuals from the islands of São Vicente, Boavista, Fogo, and Santiago. From previous reports, it was known that the islands of Santiago (70.6%), São Vicente (11.7%), Fogo (5.1%), and Boa Vista (1.5%) together account for more than 85% of the PLHIV in the country [16]. We also found that HIV-1 subtypes are unevenly distributed among the Cabo Verdean islands. The results of our study reveal that the majority of newly diagnosed participants (46.6%) were in the age group of 31 to 50 years. This age distribution is in line with previous studies carried out in the country [15,24]. The spread of this infection among young people highlights the importance of developing prevention programmes targeted to this specific group. Of the various routes of HIV transmission, the sexual route is the most common and is responsible for almost 70% of HIV-1 infections globally [27,28]. Consistently, in our study, HIV-1 infection was reported to be transmitted mainly by sexual route (91.8%). These data are consistent with other studies performed in the country and globally [29,30,31].

The HIV-1 genome presents high levels of genetic diversity and has diverged into different subtypes and recombinant forms. In this study, it was possible to identify the presence of five distinct HIV-1 pure subtypes (A, B, C, F, and G), as well as of a circulating recombinant form (CRF02_AG). The recombinant form CRF02_AG (41.1%) was the most prevalent variant identified in this population, followed by subtype G (37.0%). These results are consistent with other studies conducted in the country [23,24,25], which also reported a predominance of CRF02_AG. However, the prevalence of the CRF02_AG found in our study was even higher than that reported in previous studies, indicating that CRF02_AG might be becoming dominant. These results are also consistent with the molecular profile of HIV-1 in other West African countries neighbouring Cabo Verde, namely Guinea Bissau and Senegal [32,33,34]. CRF02_AG is, in fact, one of the most prevalent recombinant HIV-1 strains globally, with an estimated proportion of 7.7% of infections worldwide [35] and a clear predominance in West African countries [36]. This pattern may be due to the early exportation of the CRF or the parental subtypes to this region of Africa after the early ignition of the HIV-1 epidemic in Central Africa. Its global prevalence may also be explained by its greater in vitro replicative capacity compared to other subtypes [37,38]. However, its recombinant origin is still debated, given reports indicating a potential pure origin for this CRF [39] and a recombinant origin for its parental subtype G.

The island of Santiago seems to be a hub for HIV-1 in Cabo Verde, as it stands out as exhibiting the broadest genetic diversity, with all the subtypes covered in this study identified there, except for subtype F, which was found exclusively on the island of São Vicente. This remarkable genetic variability observed on the island of Santiago may be associated with the fact that it is the largest island in Cabo Verde, home to more than half of the country’s population [14], and where the capital Praia is situated. Due to its socioeconomic development, it has a high influx of people from different regions of the country and other parts of the world. This population mobility may be one of the main reasons for the increased genetic diversity of HIV-1 on Santiago Island compared to other islands. Population movements have long been recognized as a major contributor to the dissemination of HIV-1 on a global scale [40,41,42].

In our study, subtype F was identified exclusively on the island of São Vicente, in line with other previous studies [24,25]. These data highlight the consistent presence of subtype F on the island over time. The exclusivity of subtype F on this island raises questions about its origin and potential transmission routes. Despite exhibiting a relatively low global prevalence (<1%) [35], subtype F has been identified in various regions, including Central Africa, South America, and Europe, in both its non-recombinant form and as part of recombinant genomes [43,44]. The detection of this specific subtype in São Vicente emphasizes the need for phylodynamic studies to understand the origin and transmission dynamics of HIV-1 subtype F in this island.

The presence of several HIV-1 subtypes considerably increases the likelihood of occurrence of intersubtype recombination events, culminating in the generation of new recombinant forms, either circulating recombinant forms (CRFs) or unique recombinant forms (URFs). The surveillance and monitoring of the genetic evolution of HIV-1 are essential for understanding and addressing the complex issues posed by its diversity, as well as for developing effective prevention and treatment approaches in the ongoing fight against the HIV/AIDS pandemic. This study confirmed a significant association (*p* < 0.05) between the distribution of viral subtypes and the island of residence of the infected individuals. The high level of tourism in Cabo Verde and the associated migratory flows may serve as potential routes for the introduction of different variants of the virus. In addition, the country’s geographical location, as a point of intersection between the continents of Europe, America, and Africa, raises the possibility that the country may be a hub with an inflow of HIV-1 subtypes from these different geographic regions. Further studies are needed to better understand these phenomena.

This study found a moderate prevalence (9.6%) of TDR in Cabo Verde, according to the definition of the WHO [45]. However, this value is higher than that found in the last study conducted in the country (3.4%) [24], and follows the trend observed in many countries in sub-Saharan Africa and in resource-limited countries, where TDR levels exceed 10% [34,46]. The rapid increase in TDR in these countries is caused by the ARV scale-up without the associated routine surveillance of TDR in newly diagnosed patients and without resources to test for ARV DR in patients failing therapy. These factors can contribute to the persistence of high viral loads in patients left without a diagnosis of treatment failure, which can then be transmitted to other patients.

The prevalence of mutations associated with NRTIs and NNRTIs was 2.75% (n = 2) and 9.7% (n = 7), respectively. Among the most prevalent mutations, K103N was highlighted among the NNRTIs and M184V among the NRTIs. These mutations were also identified in other studies conducted in country [23,24] and in several countries in West Africa and around the world [47,48]. These data are particularly concerning as the K103N mutation is a non-polymorphic genetic mutation that confers resistance to NNRTIs, such as NVP and EFV [49,50,51]. Studies have shown that this mutation can reduce susceptibility to NVP and EFV by approximately 50 and 20 times, respectively [52,53]. It is important to highlight that K103N is the most frequently observed and transmitted drug resistance mutation [54]. On the other hand, the M184V mutation is associated with high-level resistance to 3TC and FTC, resulting in a susceptibility reduction to these drugs of more than 200 times [55,56]. While DRMs to NNRTIs are not so worrying, as this drug class is barely used currently in the country, M184V in particular should be monitored closely, since it confers resistance to drugs that are currently used as the first-line regimen in Cabo Verde. The currently used first-line regimen is TDF + 3TC + DTG. Baseline resistance to one of the NRTIs in the regimen—in this case, 3TC—can threaten the efficacy of DTG, which is then used in a context comparable to dual therapy.

When comparing our data with studies conducted in neighbouring countries, namely Senegal and Guinea-Bissau [33,57,58], we observed a high rate of TDR. According to these authors, this resistance can accelerate the spread of resistant strains, compromising the efficacy of available therapies and associating these mutations with high rates of virological failure. These studies demonstrate that continuous surveillance is essential to ensure the success of treatment and prevent the spread of resistant strains. TDR to HIV-1 not only compromises the efficacy of treatments, but also presents significant challenges for controlling the epidemic. Therefore, it is imperative that public health policies in Cabo Verde consider this evidence when developing treatment interventions, aiming to optimize clinical outcomes and contain the spread of the virus.

No mutations associated with PIs and INSTIs were detected. This absence may be related to the low use of these ART classes in the currently recommended treatment regimens in the country. With the widespread introduction of DTG and the second-line use of PIs, the emergence of resistance mutations to these drug classes may occur.

Finally, the obtained results indicated the absence of significant associations between sociodemographic and clinical variables and the presence of mutations associated with ARV resistance (Table 3). These findings suggest that such variables do not play a significant role in the occurrence of resistance mutations. It is important to note that the sample size was relatively small, which may have limited the detection of statistically relevant differences. Therefore, future studies with a larger sample have the potential to provide a deeper understanding of this issue.

Routine TDR monitoring is crucial to assess the emergence and spread of DRMs. Due to the increasing resistance to NNRTIs, the WHO recommended the use of DTG as the first-line treatment regimen for people living with HIV [21]. In addition to providing effective viral suppression, it presents a high genetic barrier to the development of HIV resistance, making it a crucial choice in the treatment of this condition [59,60,61]. In Cabo Verde, since 2019, the introduction of DTG into the first-line treatment regimen, along with lamivudine and tenofovir, has been gradually implemented. This measure is already in place in the country. However, in order to ensure the sustained effectiveness of this regimen, it is crucial to implement an effective routine system for epidemiological surveillance of antiretroviral resistance. Otherwise, the use of this drug class as first-line treatment could be threatened in the short term by the emergence of DRMs.

## 5. Conclusions

The genetic diversity of HIV-1 in Cabo Verde is dominated by CRF02_AG and subtype G. The prevalence of TDR in the country is close to 10%, which may impact the effectiveness of treatment. No mutations associated with the PI and INSTI classes were identified, suggesting that antiretroviral regimens from these classes remain effective in the country. However, given the predicted increase in drug resistance to first-line drug classes, the implementation of routine HIV drug resistance surveillance is crucial to ensure the continued effectiveness of these drugs.

## Figures and Tables

**Table 1:** Sociodemographic, behavioural, and clinical characteristics of 73 drug-naïve individuals distributed according to the HIV-1 viral subtype. The *p*-values were obtained using the chi-square test and Fisher’s exact test.

**Participant Characteristics**	**Total**	**Subtype CRF02_AG**	**Subtype G**	**Other Subtypes**	***p* Value (95%)**
n (%)	73 (100%)	30 (41.1%)	27 (37.0%)	16 (21.9%)	
Sex					
Female	35 (47.9%)	11 (15.1%)	15 (20.5%)	9 (12.3%)	0.273
Male	38 (52.1%)	19 (26.0%)	12 (16.4%)	7 (9.6%)	
Age group (years)					
≤30	13 (17.8%)	6 (8.2%)	5 (6.8%)	2 (2.7%)	0.417
31–40	17 (23.3%)	5 (6.8%)	10 (13.7%)	2 (2.7%)	
41–50	17 (23.3%)	9 (12.3%)	5 (6.8%)	3 (4.1%)	
51–60	15 (20.5%)	7 (9.6%)	4 (5.5%)	4 (5.5%)	
>60	11 (15.1%)	3 (4.1%)	3 (4.1%)	5 (6.8%)	
Island of residence					
Santiago	55 (75.3%)	23 (31.5%)	24 (32.9%)	8 (11.0%)	0.014
Other island	18 (24.7%)	7 (9.6%)	3 (4.1%)	8 (11.0%)	
Occupation					
Employee	64 (87.7%)	26 (35.6%)	23 (31.5%)	15 (20.5%)	0.922
Unemployed	6 (8.2%)	3 (4.1%)	2 (2.7%)	1 (1.4%)	
Student	3 (4.1%)	1 (1.4%)	2 (2.7%)	0 (0.0%)	
Marital status					
Married	18 (24.7%)	6 (8.2%)	9 (12.3%)	3 (4.1%)	0.503
Single	55 (75.3%)	24 (32.9%)	18 (24.7%)	13 (17.8%)	
Education level					
Illiterate/only literate	13 (17.8%)	4 (5.5%)	3 (4.1%)	6 (8.2%)	0.254
Elementary School	36 (49.3%)	16 (21.9%)	13 (17.8%)	7 (9.6%)	
Secondary Education	24 (32.9%)	10 (13.7%)	11 (15.1%)	3 (4.1%)	
Mode of Transmission					
Heterosexual	67 (91.8%)	29 (39.7%)	25 (34.2%)	13 (17.8%)	0.249
Men who have sex with men	5 (6.8%)	1 (1.4%)	2 (2.7%)	2 (2.7%)	
Other	1 (1.4%)	0 (0.0%)	0 (0.0%)	1 (1.4%)	
TDR					
Yes	7 (9.6%)	4 (5.5%)	3 (4.1%)	0 (0.0%)	0.408
No	66 (90.4%)	26 (35.6%)	24 (32.6%)	16 (21.9%)	
CD^4+^ T-cell count at diagnosis					
Mean CD^4+^ cell	344.1 ± 248.7				
<350	34 (46.6%)	14 (19.2%)	11 (15.1%)	9 (12.3%)	0.509
350–499	13 (17.8%)	5 (6.8%)	6 (8.2%)	2 (2.7%)	
≥500	13 (17.8%)	3 (4.1%)	6 (8.2%)	4 (5.5%)	
Unknown	13 (17.8%)	8 (11.0%)	4 (5.5%)	1 (1.4%)	
Viral load at diagnosis (log10 HIV-1 RNA copies/mL) IQR					
Median viral load	51,700				0.964
≤4.0	14 (19.2%)	6 (8.2%)	6 (8.2%)	2 (2.7%)	
4.1–5.0	16 (21.9%)	6 (8.2%)	6 (8.2%)	4 (5.5%)	
≥5.1	21 (28.8%)	10 (13.7%)	6 (8.2%)	5 (6.8%)	
Unknown	22 (30.1%)	8 (11.0%)	9 (12.3%)	5 (6.8%)	
Patients with co-infections					
Yes	33 (45.2%)	16 (21.9%)	11 (15.1%)	6 (8.2%)	0.542
No	40 (54.8%)	14 (19.2%)	16 (21.9%)	10 (13.7%)	

**Table 2:** Impact of drug resistance mutations identified in genomic sequences with TDR on susceptibility to Nucleoside Reverse Transcriptase Inhibitors (NRTIs) and Non-Nucleoside Reverse Transcriptase Inhibitors (NNRTIs), as analyzed using the algorithm on the Stanford HIV database.

HIV-1 Subtype	RT Mutations		Nucleoside Reverse Transcriptase Inhibitors					Non-Nucleoside Reverse Transcriptase Inhibitors				
	NRTIs	NNRTIs	ABC	AZT	FTC	3TC	TDF	DOR	EFV	ETR	NVP	RPV
CRF02_AG	None	K103N	S	S	S	S	S	S	HLR	S	HLR	S
G	None	K101E, E138Q, G190A	S	S	S	S	S	LLR	HLR	IR	HLR	HLR
CRF02_AG	None	Y181C	S	S	S	S	S	PLR	IR	IR	HLR	IR
CRF02_AG	None	K103N	S	S	S	S	S	S	HLR	S	HLR	S
CRF02_AG	M184V	K101E, K103N, G190GA	LLR	S	HLR	HLR	S	LLR	HLR	IR	HLR	HLR
G	M184V, T215Y	K103N, E138A	LLR	IR	HLR	HLR	S	S	HLR	PLR	HLR	LLR
G	None	K101E, E138Q, G190A	S	S	S	S	S	LLR	HLR	IR	HLR	HLR

Legend: NRTIs—Nucleoside Reverse Transcriptase Inhibitors; NNRTIs—Non-Nucleoside Reverse Transcriptase Inhibitors; RT—Reverse Transcriptase. Susceptible—S; Potential Low-Level Resistance—PLR; Low-Level Resistance—LLR; Intermediate Resistance—IR; High-Level Resistance—HLR. ABC (abacavir), AZT (zidovudine), FTC (emtricitabine), 3TC (lamivudine), and TDF (tenofovir), DOR (doravirine), EFV (efavirenz), ETR (etravirine), NVP (nevirapine), and RPV (rilpivirine).

**Table 3:** Factors associated with the occurrence of drug resistance mutations based on logistic regression analysis. Ref—reference category; OR—Odds Ratio. Other islands include São Vicente, Fogo, and Boa Vista, and other modes of transmission include blood transfusions. * *p*—values were obtained using the chi-square test and Fisher’s test.

Variable	With TDR	Without TDR	* *p* Value	OR (95%CI)	*p* Value
	Frequency n = 7 (9.6%)	Frequency n = 66 (99.4%)			
Gender					
Female	3 (4.1%)	32 (43.8%)	1.000	Ref	0.777
Male	4 (5.5%)	34 (46.6%)		1.255 (0.26–6.049)	
Age group (years)					
≤30	4 (5.5%)	9 (12.3%)	0.037	Ref	
31–40	0 (0.0%)	17 (23.3%)		0.00 (0.00–0.00)	0.998
41–50	1 (1.4%)	16 (21.9%)		0.141 (0.014–0.01)	0.100
51–60	2 (2.7%)	13 (17.8)		0.346 (0.05–2.31)	0.273
>60	0 (0.0%)	11 (15.1)		0.00 (0.00–0.00)	0.999
Island of residence					
Other islands	2 (2.7%)	16 (21.9%)	0.801	Ref	0.801
Santiago	5 (6.8%)	50 (68.5%)		0.8 (0.14–4.53)	
Occupation					
Employee	5 (6.8%)	59 (80.8%)	0.205	Ref	
Unemployed	1 (1.4%)	5 (6.8%)		2.36 (0.23–24.33)	0.471
Student	1 (1.4%)	2 (2.7%)		5.9 (0.45–76.95)	0.176
Marital status					
Single	6 (18.2%)	49 (67.1%)	0.673	Ref	0.511
Married	1 (1.4%)	17 (23.3%)		0.48 (0.05–4.28)	
Education Level					
Secondary Education	2 (2.7%)	22 (30.1%)	0.448	Ref	
Elementary School	5 (6.8%)	31 (42.5%)		1.774 (0.315–9.992)	0.516
Illiterate/only literate	0 (0.0%)	13 (17.8%)		0.00 (0.00–0.00)	0.999
Mode of transmission					
Heterosexual	7 (9.6%)	60 (82.2%)	1.000	Ref	
Men who have Sex with Men	0 (0.0%)	5 (6.8%)		5.167 (0.406–65.677)	0.210
Other	0 (0.0%)	1 (1.4%)		0.00 (0.00–0.00)	0.999
CD^4+^ T cell count at diagnosis					
<350	4 (5.5%)	30 (41.1%)	0.792	Ref	
350–499	1 (1.4%)	12 (16.4%)		0.625 (0.063–6.180)	0.688
≥500	0 (0.0%)	13 (17.8%)		0.00 (0.00–0.00)	0.999
Unknown	2 (2.7%)	11 (15.1%)		1.364 (0.218–8.523)	0.740
Subtype					
HIV-1 subtype G	3 (4.1%)	24 (32.9%)	0.408	Ref	
CRF02_AG recombinant form	4 (5.5%)	26 (35.6%)		1.231 (0.25–6.07)	0.799
HIV-1subtype others	0 (0.0%)	16 (21.9%)		0.00 (000)	0.998

## Data Availability

The original contributions presented in this study are included in the article. Further inquiries can be directed to the corresponding author.
